# Reviews

**Published:** 1881-12

**Authors:** 


### Reviews.

**A Text-Book of Physiology.** By M. FOSTER, M. A., M. D., F. R. S. Second American edition, by EDWARD T. REICHERT, M. D. Philadelphia: Henry C. Lea's Son & Co.

The readers of the former American edition will, doubtless, agree that there is no better text book on physiology than this, in print. Besides being clear and simple in its statements, it is abreast with the very latest researches, and these are presented in an instructive and, indeed, in an entertaining style. Comparison is not criticism, and we, therefore, avoid the mention of other standard works, which have long stood before the student as oracles in physiology. It is certain, however, that Foster presented to us the most recent phases of the subject in a very attractive light, and the additions of Dr. Reichert are such as to elucidate any points which might possibly have seemed obscure. In a brief notice of any book it is evidently impossible to review critically its various parts, but we can not omit a word of unusual praise of the excellent manner in which the author treats the "special senses." In general it is a most admirable work, and this new edition of it will prove acceptable, not only to younger students, but also to physicians who still wish to base their treatment of the abnormal on a knowledge of normal conditions of the body.

**Favorite Prescriptions of Distinguished Practitioners; with Notes on Treatment.** By B. W. PALMER, A. M., M. D. New York: Birmingham & Co.

A small book which requires an equally small space for its notice. As an enumeration of formulæ employed, it may be a success, or for one who cares to follow a certain rut without variation, it may be convenient; but as a guide to the rational treatment of disease which changes its form in almost every case, it is necessarily a failure. Unfortunately, however, many practitioners will search for a prescription, more assiduously than for an intelligent diagnosis and among such the manual will probably find a ready sale.

---

**The Medical Record Visiting List for 1882.** New York: Wm. Wood & Co.

This is certainly the most handsome, if not the most concise and compact of all the lists published. It contains all that is necessary in a pocket memorandum of professional visits, and the arrangement is good. We find that in this city the Medical Record List is steadily growing in favor.

---

**The Physician's Memorandum Book.** Arranged by JOEL A. MINER, Ann Arbor, Mich. Fifth improved edition. Price, $1.25.

This new Visiting List has all the general advantages of books of this class. The publisher lays claim to superiority in the general convenience of its blanks, its ledger sheets and its cash account.

---

**Walsh's Call-Book and Handy Ledger.** RALPH WALSH, M. D., Washington, D. C.

These two books are well adapted to the wants of the practitioner. The Call-Book, or Visiting List, has special advantages of its own, which give it a high place in the consideration of a great many physicians. The use of the Handy Ledger will enable even the busiest physician to keep his own accounts with the least possible expenditure of time and trouble.

